# Professional burnout syndrome: Alexithymia, empathy and communication mechanismsи

**DOI:** 10.1192/j.eurpsy.2021.939

**Published:** 2021-08-13

**Authors:** N. Chernus, K. Serdakova, I. Vasilyeva, S. .Sivkov, T. Savina, A. Sivkov

**Affiliations:** 1 Department Of Outpatient Therapy, I.M. Sechenov First Moscow State Medical University: Moscow, Russia, Moscow, Russian Federation; 2 Department Of Psychology, I.M. Sechenov First Moscow State Medical University: Moscow, Russia, Moscow, Russian Federation; 3 The Department Of Clinical Pharmacology And Internal Diseases Propaedeutics, the I.M. Sechenov First Moscow State Medical University, Moscow, Russian Federation

**Keywords:** burnout, adaptation, empathy, alexithymia

## Abstract

**Introduction:**

Professional burnout syndrome (PBS) is currently considered from the perspective of value-oriented sense –of underlying psychological factors contributing to PBS development promotes the relevance of this study.

**Objectives:**

The study population included 81 medical professionals from out-patient polyclinic healthcare institution, among which 47(58%) healthcare professionals showed symptoms of burnout (mean age – 38,5±11,4 years old).

**Methods:**

‘Attitude to Work and Professional Burnout’ by V.A. Vinokur, ‘Coping Strategies’ by S. Folkman and R. Lazarus Р., Spielberg’s Questionnaire; TAS-26; Emotional Response Scale by A. Megrabyan and N. Epstein.

**Results:**

The correlation analysis revealed certain interdependencies between the professional burnout symptoms and personal qualities of subjects. Thus, the higher burnout level correlated with increased emotional burnout (r=0,871; p=0,016), reduced professional satisfaction (r=0,624; p=0,031), poorer health and adaptation (r=0,872; p=0,023), increased state anxiety (r=0,551; p=0,000), increased alexithymia scores (r=0,823; p=0,017); reduced empathy scores as emotional response to others’ emotional experience (r=0,466; p=0,000) and reduced willingness to involve into other people’s issues (r=0,564; p=0,032). No statistically significant correlations between TAS total alexithymia score and empathy score were demonstrated.

**Conclusions:**

The healthcare professionals employed at the out-patient polyclinic units belong to the at-risk population group of professional burnout syndrome development. The individuals with higher burnout levels show typical specific correlations of empathy forms: in particular, decreased ability to differentiate one’s feelings from feelings of others, increased emotional sensitivity and reduced willingness to involve into other people’s issues are usually observed.

